# Hierarchical Local-Global Feature Fusion Network for Robust Ship Target Recognition in Complex Maritime Environment

**DOI:** 10.3390/s26010029

**Published:** 2025-12-19

**Authors:** Xuanhe Liu, Shuning Zhang, Si Chen, Jianchao Li, Yingying Luo

**Affiliations:** School of Electronic and Optical Engineering, Nanjing University of Science and Technology, Nanjing 210094, China; xuanheliu@njust.edu.cn (X.L.); chensi354@njust.edu.cn (S.C.); lijianchao@njust.edu.cn (J.L.); 124104010614@njust.edu.cn (Y.L.)

**Keywords:** ship target recognition, local-global feature integration, hybrid model, self-attention representation, maritime scene analysis, limited sample learning

## Abstract

**Highlights:**

**What are the main findings?**

- This paper proposes a hierarchical local-global feature fusion model that integrates local structural features extracted by convolutional neural networks with global semantic dependencies modeled by Transformer architectures through a progressive multilayer self-attention mechanism.
- Extensive experiments on both the FUSAR dataset and a measured dataset demonstrate that the proposed model achieves superior classification accuracy and F1 scores compared with traditional CNNs, pure Transformer models, and representative recent vision architectures, while maintaining competitive inference efficiency. The model also exhibits strong robustness under low signal-to-noise ratios and limited sample conditions.

**What are the implications of the main findings?**

- Hierarchical encoding of local structural features and global contextual dependencies provides a novel approach for extracting vessel target features under complex sea conditions, enhancing the reliability of maritime target recognition.
- Transfer learning methods based on partial fine-tuning can efficiently adapt to limited labeled data, enabling rapid deployment of high-precision recognition systems in resource-constrained environments.

**Abstract:**

Accurate ship target recognition remains challenging in complex maritime environments due to background clutter, multiscale target appearance, and limited discriminative features extracted by single-type networks. To address these issues, this paper proposes a hierarchical local-global feature fusion network (HLGF-Net) that integrates local structural cues from a CNN encoder with global semantic dependencies modeled by a Transformer. The proposed model progressively constructs hierarchical dependencies through stacked Transformer blocks, enabling comprehensive integration of local structural details and global semantic context. This design enhances the capability to capture fine-grained local contours and long-range global contextual relationships simultaneously. Extensive experiments on ship recognition datasets demonstrate that HLGF-Net achieves superior performance compared with traditional CNNs, pure Transformers, and representative recent vision architectures, particularly under conditions of cluttered backgrounds, partial occlusion, and limited target samples. The proposed framework provides an effective solution for robust maritime target recognition and offers a general strategy for hierarchical local-global feature integration.

## 1. Introduction

Maritime target recognition plays a critical role in ocean surveillance, maritime security, and rescue missions [1,2]. However, accurate ship recognition remains challenging because sea clutter, originating from radar backscattering of the dynamic sea surface, often masks target echoes, while target appearance variations and the limited availability of labeled samples in real maritime environments further exacerbate this difficulty [3,4,5]. Radar-based ship recognition systems are therefore particularly vulnerable to background interference and low signal-to-noise ratio (SNR) conditions, which significantly degrade classification performance [6]. Recent studies further indicate that small ships embedded in heavy sea clutter require more discriminative feature extraction mechanisms to avoid severe performance degradation [7].

Early maritime radar target studies primarily focused on target detection and sea clutter suppression using traditional signal processing techniques, such as wavelet-based transformations and handcrafted feature extraction methods [5,8,9,10,11], resulting in limited capability for target recognition under complex maritime environments. With the rapid development of deep learning, convolutional neural networks (CNNs) have been widely applied to ship detection and recognition tasks [12,13]. Multiscale and hierarchical convolutional feature learning strategies have shown improved robustness under complex maritime environments [7,14,15]. In addition, fusion strategies exploiting complementary SAR perspectives have been investigated to enhance spatial consistency and robustness in cluttered maritime scenes [16]. Despite these advances, CNN-based models rely primarily on local receptive fields and struggle to capture long-range dependencies, which limits their discriminative capability when targets exhibit similar local structures or are heavily corrupted by clutter [17,18]. Deep neural networks have also been widely applied in radar signal processing under complex maritime electromagnetic environments, demonstrating strong adaptability to interference and non-stationary signals [19,20,21,22].

At the same time, earlier studies on deep convolutional neural networks emphasized the importance of network depth and hierarchical feature representations, leading to the development of architectures such as AlexNet, VGG, and GoogLeNet [23,24,25,26]. In recent years, the Transformer architecture [27], originally developed for natural language processing, has been introduced into the field of computer vision tasks through the self-attention mechanism [28]. Compared with CNNs, Transformers excel at modeling global contextual dependencies and have demonstrated outstanding performance in various recognition tasks. However, pure Transformer models typically require large-scale training data and exhibit unstable performance under the conditions of small sample sizes or low signal-to-noise ratios, which are common in maritime scenarios.

In order to leverage the complementary strengths of CNNs and Transformers, several hybrid architectures have been proposed for ship recognition and related remote sensing tasks [12,14,15]. However, most existing hybrid methods employ Transformers only as a terminal global aggregation module applied to the final convolutional feature map. Such designs lack an explicit mechanism for progressively integrating local structural features with global semantic dependencies across network depth, particularly under limited sample conditions.

Beyond maritime recognition, robust feature modeling and information fusion under degraded signal conditions have also been investigated in related sensing and positioning fields, such as GNSS-assisted cooperative localization and distributed navigation systems [29,30,31,32]. These studies further show the importance of effective local–global information integration in complex and clutter-dominated environments.

To reduce the reliance on large labeled datasets, researchers have also explored the application of weakly supervised learning strategies, such as pseudo-label optimization, in maritime target recognition [33]. However, most existing methods primarily focus on detection tasks or coarse recognition and exhibit limited capability in detailed ship type classification [13]. Therefore, we propose a hierarchical local–global feature fusion network (HLGF-Net) for robust ship target recognition in complex maritime environments. The proposed model integrates convolutional neural networks for local structural feature extraction with a stacked Transformer encoder for global semantic modeling, enabling progressive deep interaction between local and global representations. By explicitly constructing hierarchical local–global dependencies, HLGF-Net enhances feature discriminability under limited sample and low SNR conditions. Extensive experiments conducted on both a public SAR dataset and a measured maritime dataset demonstrate that the proposed method achieves superior recognition accuracy and robustness compared with several representative baselines.

## 2. Methods

### 2.1. Overall Framework

**Figure 1:**
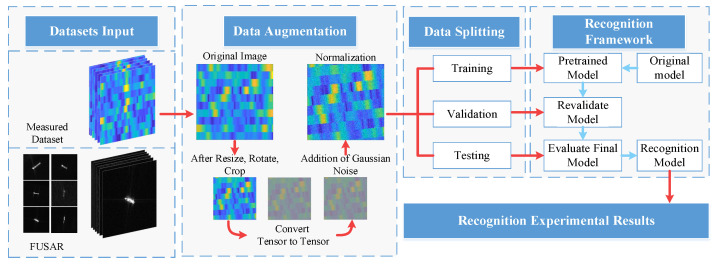
Overall framework of the proposed HLGF-Net.

Recognizing different categories and instances of ship targets under the same sea clutter environment remains a challenging problem. Based on these considerations, this study proposes a ship target recognition framework, which is evaluated using both public SAR datasets and measured data collected from Yantai experiments to verify its robustness and effectiveness under complex sea clutter conditions, as illustrated in Figure 1. The red arrows within Figure 1 indicate the sequence of data processing steps, where the blue arrows indicate the specific operations applied to the input data within the network.

Within this framework, under limited sample conditions, transfer learning and fine-tuning are commonly adopted to improve model generalization in maritime target recognition. In this study, the recognition network employs a pretrained VGG backbone initialized with ImageNet weights as a local feature extractor, where most convolutional layers are retained, and the original fully connected layers are removed. The resulting convolutional feature maps are then forwarded to the subsequent Transformer blocks for global contextual modeling and final ship target classification.

### 2.2. Datasets and Data Preparation

#### 2.2.1. Datasets

In this study, partial slices from an open SAR imaging dataset (FUSAR) and measured data collected from the Yantai area are used for maritime ship target recognition experiments. Due to differences in sample characteristics and data volume, only samples satisfying the experimental requirements are selected and preprocessed before use. Due to the direct impact of sample volume on the recognition accuracy of the network in target identification tasks, we address the issue of unequal data volumes within the dataset. Some target categories contain relatively fewer samples. We select suitable data samples for our experiments based on the characteristics of each dataset and perform corresponding data preprocessing to construct the dataset for the recognition network.

The FUSAR dataset provides 512 × 512 grayscale SAR image chips, from which three categories, BulkCarrier (546 samples), CargoShip (630 samples), and Fishing (785 samples), were selected to assess the network’s capability in recognizing scenarios with relatively abundant intraclass samples. A careful sample selection process followed by data augmentation was implemented to equalize the sample count across these classes, suitably representing conditions typical of smaller datasets.

In our experimental setup, the measured dataset comprises vessel samples derived from imaging data. Owing to the inherent complexity of the task and the extended duration of individual experiments, acquiring substantial data on vessels across diverse categories is often constrained. Furthermore, considerable individual variation exists among vessels, necessitating a distinctive approach to dataset construction. Consequently, during data organization, each group of vessel data is treated as a distinct target category. This methodology ensures the specific characteristics of each vessel class are fully reflected. Although each vessel category contains approximately 200–250 samples, this scale reflects the realistic constraints of maritime SAR data acquisition. Furthermore, multiangle unfolding and augmentation strategies considerably increase intraclass variability, effectively reducing the risk of overfitting under limited sample conditions.

**Figure 2:**
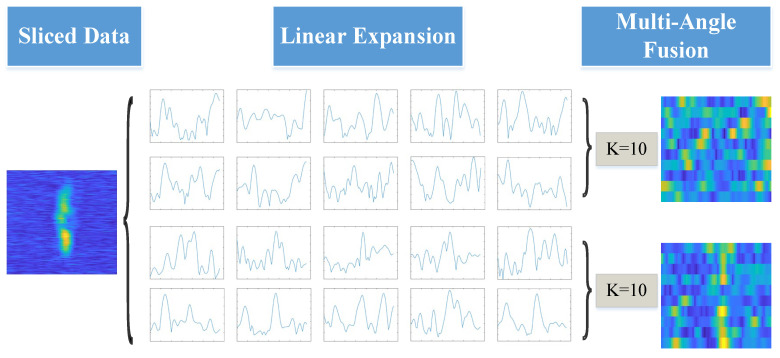
Data processing framework of measured dataset.

Following pulse compression processing of the raw data, a two-dimensional virtual mapping of target and adjacent sea surface echoes is generated. In addition, combined with real-time ship trajectory data from Shipxy, an online ship tracking platform, multiangle one-dimensional distance data of labelled ships can be extracted from the mapping. Figure 2 indicates the data construction framework of the measured dataset, including the linear unfolding of the raw data and its organization across different observation angles and range dimensions. Table 1 summarizes the detailed information of the measured dataset.

#### 2.2.2. Data Preparation and Augmentation

Following standard supervised learning practice, the dataset is divided into three mutually exclusive subsets for training, validation, and testing [34]. To preserve the original class distribution and address data imbalance, stratified sampling is employed during dataset partitioning. Specifically, samples from each category are divided into training, validation, and test sets using a 60:20:20 ratio, ensuring that each subset maintains a consistent class proportion with the overall dataset.

In this research, several data augmentation techniques are applied during preprocessing to increase data diversity. This module incorporates a comprehensive set of data augmentation techniques, intended to enhance the accuracy of sea surface target detection and classification by the model. The techniques include the following:

1. Three-channel processing of images better simulates the visual information of the real world and makes it easier for the network to find more detailed target features;
2. Dimensionality restructuring to accommodate the network input requirements;
3. Central cropping to reinforce the model’s focus on the central area of targets;
4. Random rotation to increase the diversity of data viewpoints and enable the model to adapt to different observation angles;
5. Addition of Gaussian noise to improve the model’s robustness against noise conditions;
6. Tensorization and normalization of data to ensure consistency and effectiveness of data inputs.

These methods focus on the special features of radar reflect data, especially how it is affected by the way targets are positioned and the noise that can interfere. The main goal is to improve the model’s performance by preparing the data better upfront.

### 2.3. Transfer Learning Strategy

Based on these considerations, a partial fine-tuning strategy is adopted in this work under limited sample conditions. Specifically, the network is initialized with pretrained weights, and most convolutional layers are frozen during training, while only higher-level layers are updated to adapt to the ship target recognition task, which has been shown to facilitate stable optimization and effective learning when training data are limited [35,36].

Transfer learning and the partial fine-tuning strategy adopted in this work enable the pretrained convolutional backbone to provide stable local representations, while allowing the Transformer module and classification head to adapt effectively to the target domain under limited sample conditions [37]. During training, the majority of the VGG parameters are frozen, and only the final few convolutional layers, all the Transformer modules, and the classification heads are fine-tuned. This strategy is designed to improve generalization ability, mitigate the risk of overfitting, and facilitate effective adaptation to maritime target recognition tasks with limited labeled data.

From a formal perspective, transfer learning aims to optimize the target task model by leveraging knowledge from a related source task, which can be expressed as follows [38]:

(1) $$\underset{f_{t}}{\min}L_{t}(f_{t}(x),y_{t})+\lambda R(f_{s},f_{t})$$

where $f_{t}$ denotes the model for the target task, $L_{t}$ denotes the target task loss function, λ is the regularization parameter, and $R\left(\cdot \right)$ measures the discrepancy between the source and target task models. The target task loss can be written as follows:

(2) $$L_{t}(f_{t}(x),y_{t})=\frac{1}{m}\sum_{i=1}^{m}l(f_{t}(x_{ti}),y_{ti})$$

where *m* is the number of target samples. The regularization term is defined as follows:

(3) $$R(f_{s},f_{t})=|\theta_{s}-\theta_{t}{|}^{2}$$

where $\theta_{s}$ and $\theta_{t}$ represent the model parameters of the source and target tasks, respectively.

With the above transfer learning and fine-tuning strategy, the proposed model can be efficiently trained under limited labeled data while preserving stable feature representations. Based on this training paradigm, the detailed network architecture is described in the following subsection.

### 2.4. Network Architecture of HLGF-Net

#### 2.4.1. Overview of the Network Architecture

The proposed network adopts a hybrid convolution–Transformer architecture designed around a hierarchical local–global feature fusion paradigm. Instead of explicitly aggregating multiscale or multistage convolutional features, the model relies on a pretrained VGG backbone to extract a compact and semantically rich convolutional feature map, which serves as a unified local structural representation.

After convolutional feature extraction, the resulting feature map is partitioned into a set of patch embeddings, each corresponding to a localized spatial region. These patch tokens collectively encode detailed local structural information. A learnable global classification token is then appended to the patch sequence to represent holistic semantic information. Positional embeddings are added to preserve spatial ordering before the token sequence is fed into the Transformer encoder.

Hierarchical local–global feature fusion is defined as a deep semantic integration process where local representations and a global classification representation interact progressively across multiple Transformer layers, rather than being fused through explicit multistage convolutions or single-stage feature aggregation.

In the proposed architecture, the notion of hierarchy does not originate from the convolutional backbone through multiscale feature extraction. Instead, it emerges from the progressive semantic refinement performed within the stacked Transformer encoder. Across successive self-attention layers, local patch tokens and the global token continuously exchange information, enabling local representations to become increasingly contextualized by global semantics while the global representation is iteratively refined by aggregating information from all local regions.

Unlike conventional CNN–Transformer hybrid models, in which the Transformer is typically attached as a terminal global aggregation module applied only once to the final convolutional feature map, the proposed network directly embeds local convolutional representations into a stacked Transformer encoder. As a result, global semantic representations are not produced in a single stage, but are progressively constructed through repeated local–global interactions across multiple Transformer layers. This depth-wise semantic refinement mechanism constitutes the core architectural distinction of the proposed model and enables explicit hierarchical local–global feature fusion.

**Figure 3:**
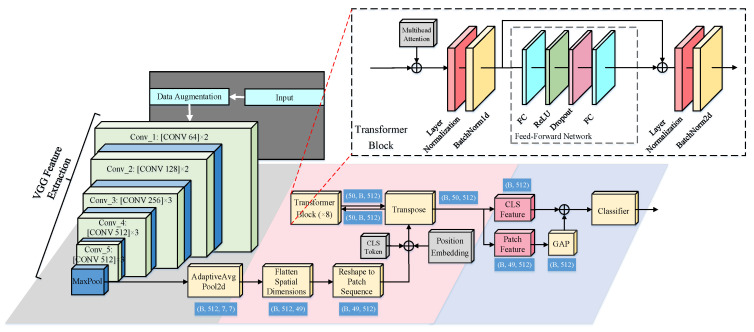
Layer structure of the proposed model.

An overview of the proposed model is shown in Figure 3. The model adopts a two-stage architecture in which a pretrained VGG backbone first produces a unified convolutional feature map representing localized structural information. This single-level feature map is then reshaped into patch embeddings and combined with a learnable classification token and positional encoding to form the token sequence that serves as the input to the Transformer encoder. The stacked Transformer layers progressively model long-range dependencies through multilayer self-attention, enabling depth-wise refinement of both local and global representations.

Different from traditional convolutional neural networks (CNNs) that focus only on the extraction of local patterns and edge information, the designed Transformer module effectively combines local features with global semantic information through the self-attention mechanism, enhancing the model’s ability to represent objects in complex scenarios. This architecture combines the VGG network’s sensitivity to fine-grained features with the Transformer’s ability to model global context, significantly improving the model’s stability and accuracy in object recognition tasks. It demonstrates outstanding robustness, particularly in electromagnetic imaging scenarios characterized by diverse object morphologies and complex environments.

Therefore, the hierarchical property of the proposed model is established entirely within the stacked Transformer encoder through progressive depth-wise semantic interactions between local patch tokens and the global classification token, rather than through multistage convolutional feature extraction.

#### 2.4.2. Hierarchical Local–Global Feature Modeling Principle

In the proposed model, the pretrained VGG backbone is used solely as a local structural encoder to generate a single unified convolutional feature map, which serves as the input to the subsequent Transformer-based semantic modeling module. Specifically, given an input image $X\in R^{H\times W\times C}$, the final convolutional stage of VGG produces a feature map $F\in R^{B\times C\times H^{\prime }\times W^{\prime }}$, where *B* denotes the batch size, and *C* denotes the channel dimension.

Suppose the input feature map is X, and the convolution kernel is $W\in R^{k\times k\times C\times D}$. The convolutional process of VGG can be expressed as follows:

(4) $$F_{i,j,d}=\sum_{m=1}^{C}\sum_{u=0}^{k-1}\sum_{v=0}^{k-1}X_{i+u,j+v,m}\cdot W_{u,v,m,d}+b_{d}$$

where k is the size of the convolution kernel, $F_{i,j,d}$ represents the position $\left(i,\;j\right)$ and channel number d within the output feature map, $X_{i+u,j+v,m}$ represents the position $\;\left(i+u,\;j+v\right)$ and channel number m within the input feature map, and $b_{d}$ is the bias term for the d-th output channel.

Although the VGG backbone contains multiple convolutional layers internally, the proposed model utilizes only its final feature map as a unified local representation. No multilevel convolutional features are extracted or fused; instead, the hierarchy is formed entirely within the Transformer depth.

The feature map extracted by the VGG backbone, denoted as $F\in R^{B\times C\times H\prime \times W\prime }$, is reshaped into a sequence of patch embeddings with length $N=H^{\prime }\times W^{\prime }$, resulting in $X\in R^{B\times N\times C}$, where each patch token $x_{b,k}\in R^{C}$ corresponds to the local feature at spatial location k (k=1,…,N) of the *b*-th sample.

To enhance the expressive capability of the sequence, a learnable classification token $z_{cls}\in R^{1\times C}$ is introduced and concatenated at the beginning of the sequence, resulting in the extended sequence:

(5) $$\tilde{X}=\left[z_{cls};\;X\in R^{B\times (N+1)\times C}\right]$$

After adding positional embeddings, the sequence $\tilde{X}$ is fed into the Transformer encoder for global contextual modeling, producing an output sequence of the same dimension:

(6) $$Z=[z_{cls},\;z_{1},\;z_{2},\ldots ,z_{N}]\in R^{B\times (N+1)\times C}$$

Hierarchical local-global feature fusion in the proposed model is therefore realized through progressive depth-wise semantic integration rather than a single-stage feature aggregation operation. In the early Transformer layers, patch tokens primarily preserve localized structural characteristics, while the global token captures global context. As the network depth increases, local representations become increasingly contextualized by global semantic information, and the global token is iteratively refined by accumulating responses from all local regions. This repeated interaction across multiple Transformer layers forms a hierarchy of semantic representations along the depth of the network.

After processing by the Transformer, the output consists of N+1 tokens, including a special classification token (CLS) and N patch tokens.

To aggregate the semantic information from all patches into a fixed-length global feature vector representing the input image, global average pooling (GAP) is applied over the patch tokens, excluding the CLS token. Specifically, the global feature vector $f_{b}\in R^{C}$ is computed as follows:

(7) $$f_{b}\;=\;\frac{1}{N}\sum_{k=1}^{N}z_{b,k}\;$$

where $f_{b}$ is the global image feature vector for the b-th sample, and *C* indicates the depth (number of channels) of the feature map. Each element in $f_{b}$ is the average value of the corresponding b-th channel of the feature map.

Consider that the patch token sequence is obtained by flattening the feature map $F\in R^{B\times C\times H\prime \times W\prime }$ in order, the mapping between sequence position k and spatial coordinates (i,j) is given by

(8) k=(i−1)×W′+j,i=1,…,H′,j=1,…,W′

The d-th channel of the global pooled feature is computed as follows:

(9) $$f_{b,d}\;=\frac{1}{H\prime W\prime }\sum_{i=1}^{H\prime }\sum_{j=1}^{W\prime }Z_{b,k,d},k=(i-1)W\prime +j,d=1,\ldots ,C$$

Finally, after the final Transformer block, the output sequence consists of the transformed classification token $z_{cls}\in R^{C}$ and 49 patch feature embeddings, each of dimension *C* (here *C* = 512). To aggregate these features for classification tasks, we perform the following steps:

- Extracting the classification token $z_{cls}$, which serves as a global representation of the input;
- Computing the global average pooling (GAP) over the 49 patch embeddings to obtain a pooled feature vector $f_{b}$;
- Concatenating the classification token and the pooled feature to form the final feature vector:

(10) $$h_{b}\;=[z_{cls};\;f_{b}\;]\in R^{2C}$$

This concatenated feature vector $h_{b}$ has dimension 1024 and is then subsequently fed into a fully connected layer for classification.

Although the final fusion occurs only at the top layer, the fused features representation originates from the unified convolutional representation extracted by the backbone and the progressively refined semantic representations produced by the multilayer Transformer encoder. This ensures that the proposed model effectively integrates the unified local information extracted by the convolutional backbone with the global semantics progressively modeled by the multilayer Transformer. This design explicitly integrates unified local structural responses extracted by the convolutional backbone with progressively refined global semantic representations produced by the multilayer Transformer encoder, resulting in a depth-wise hierarchical local-global feature fusion framework.

## 3. Results

### 3.1. Experimental Setup

In order to ensure fair and reproducible evaluation of both recognition performance and computational efficiency, all experiments were conducted under uniform hardware and software configurations.

Specifically, all models were trained and evaluated on a workstation equipped with an NVIDIA RTX 5060 Ti GPU and an Intel Core i5-12600KF processor. The implementations were developed using the PyTorch framework (version 2.9.1) with CUDA support. All models employed identical input resolutions, batch sizes, and optimization strategies to ensure consistency and fairness. Unless otherwise specified, all models were trained using the Adam optimizer with an initial learning rate of 1 × 10^−4^, a batch size of 16, and identical training schedules.

A standardized timing protocol was established to evaluate the computational cost of each model. Inference time was measured only based on the forward pass execution of the network, explicitly excluding data loading and input preprocessing overhead. To mitigate the impact of asynchronous execution, the network execution was synchronized with the GPU immediately before and after forward propagation. Multiple warm-up iterations were performed prior to formal timing to stabilize the GPU performance. The reported inference time corresponds to the average latency per sample, computed by accumulating the total forward pass time over all batches and dividing by the total number of samples.

Similarly, the training time of each sample was measured in a clearly defined manner, encompassing forward propagation, backward propagation, and parameter update steps within the training loop. The average training time per sample was obtained by dividing the total training time by the number of processed training samples, ensuring a fair comparison of computational efficiency across different architectures.

All baseline models, including LeNet, ResNet50, VGG, Vision Transformer (ViT), and the proposed method, were evaluated using the same experimental protocol, guaranteeing a reliable and reproducible comparison. This protocol ensures that differences in measured inference and training times reflect inherent computational characteristics of the models, rather than variations in implementation or experimental settings.

In summary, the adopted experimental setup and timing methodology provide a rigorous and consistent framework for evaluating the classification performance and computational efficiency of different models, enabling fair and reproducible comparisons in maritime target recognition tasks.

### 3.2. Overall Performance Comparison

This study presents a comparative analysis of several mainstream models in terms of classification performance and computational efficiency on the FUSAR public dataset. The evaluation included traditional models such as LeNet [39], VGG, and ResNet50 [40], as well as more recent architectures ViT, Swin Transformer Tiny (Swin-T), ConvNeXt Tiny (ConvNeXt-T), and the proposed HLGF-Net. For each model, we recorded final test accuracy, F1 score, parameter sizes, FLOPs, inference time per sample, and training time per sample, as summarized in Table 2.

In terms of inference latency, the proposed model achieves an average inference time of 3.902 ms per sample, which is comparable to that of VGG (3.965 ms) and Swin-T (3.368 ms), and slightly higher than ConvNeXt-T (2.859 ms), and substantially lower than ViT (23.931 ms). This indicates that the introduction of Transformer-based global feature modeling does not substantially increase inference latency when combined with the VGG backbone, and the proposed model maintains practical efficiency despite the added complexity.

In contrast, although the proposed model has a higher parameter size and FLOPs than most baseline models, its inference and training times remain competitive while achieving the highest recognition capabilities. Specifically, it outperforms VGG, ResNet50, Swin-T, and ConvNeXt-T in terms of recognition accuracy and F1 score, while keeping computational cost at a reasonable level. By comparison, ViT exhibits much higher inference latency due to the computationally intensive self-attention mechanism, while LeNet achieves low latency at the cost of significantly reduced recognition accuracy.

Overall, the results show that the proposed HLGF-Net achieves the highest recognition performance, with a final test accuracy of 91.35% and an F1 score of 0.9130, while maintaining competitive training and inference efficiency. This balance between performance and computational cost highlights its suitability for maritime target recognition tasks, even when compared with both traditional and modern architectures.

### 3.3. Robustness Evaluation Under Different SNR Conditions

In addition to evaluating performance on clean data, robustness to noise interference is also essential for maritime SAR applications. Therefore, an SNR-based experiment is conducted to assess the proposed model under varying noise conditions. Additive Gaussian noise with different signal-to-noise ratios is injected into the input samples to simulate the noise interference experienced by target echo signals under various SNR conditions. The noise variance is determined according to the predefined SNR level by first computing the signal power of each data sample and then adjusting the noise power accordingly. This approach follows standard practices in signal processing, where additive Gaussian noise is widely used to simulate low SNR conditions.

**Figure 4:**
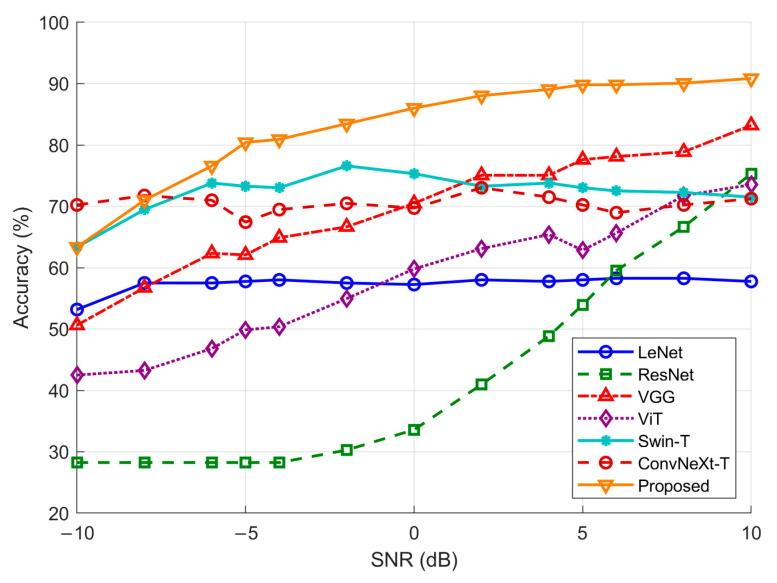
Model accuracy at different signal-to-noise ratios.

To further evaluate the performance of the proposed model under such conditions, HLGF-Net was compared with seven representative models, including LeNet, Resnet50, VGG, ViT, Swin-T, and ConvNeXt-T. The classification accuracies across different SNR conditions are presented in Figure 4. Overall, the proposed model consistently achieves the highest recognition accuracy over the entire SNR range and exhibits the slowest performance degradation trend under noise enhancement conditions.

In contrast, the baseline models exhibit clear performance differences across SNR conditions. LeNet and ResNet degrade sharply at low SNRs (below −5 dB), indicating limited robustness to heavily corrupted inputs. Traditional CNNs such as VGG remain relatively stable at medium-to-high SNRs but still deteriorate faster than the proposed model when noise increases. Among advanced architectures, ViT shows pronounced sensitivity to low-SNR noise because it relies heavily on global representations, whereas Swin-T and ConvNeXt-T benefit from local attention or convolutional priors and achieve better stability over a broader SNR range. Nevertheless, none surpasses the proposed HLGF-Net. As shown in Figure 4, the proposed HLGF-Net maintains higher recognition accuracy across all SNR levels compared with the baseline models.

### 3.4. Confusion Matrix Analysis

To provide a more intuitive validation of different models’ performance in classification tasks, this study further analyzed the prediction results of each network on the FUSAR and the Measured Dataset. Confusion matrices were employed to visually demonstrate their classification effectiveness. These matrices clearly reflect the models’ classification accuracy and error rates across different categories, thereby enabling a more comprehensive assessment of their suitability for practical applications. The confusion matrices for each network are shown below. Comparative analysis further reveals the practical advantages in classification capability demonstrated by the model proposed in this paper.

**Figure 5:**
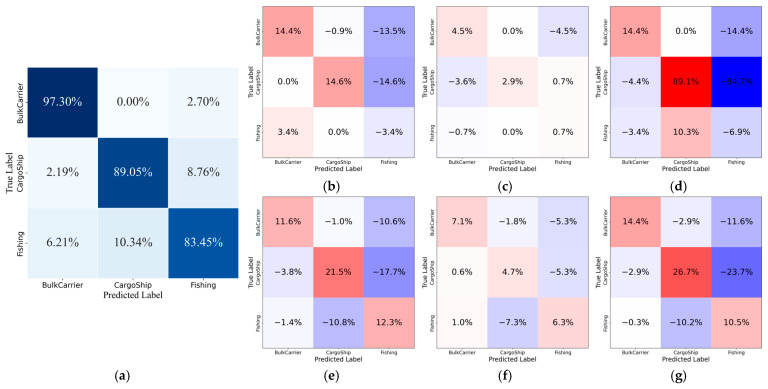
Confusion matrix of the proposed HLGF-Net on the FUSAR dataset and the corresponding difference heatmaps compared with baseline models: (**a**) Confusion matrix of the proposed model. (**b**–**g**) Normalized confusion matrix difference heatmaps between the proposed model and VGG, ResNet50, LeNet, Swin-T, ViT, and ConvNeXt-T, respectively. Positive and negative values indicate the increase and decrease in classification probability of the proposed model relative to the compared methods.

Figure 5 further compares the confusion matrices of the proposed HLGF-Net with those of several baseline models on the FUSAR dataset, providing a detailed comparison of classification behavior across the three ship categories. It can be observed that the proposed model achieves significantly higher recognition accuracy for the BulkCarrier and CargoShip categories than any other baseline models. These improvements indicate that the joint modeling of local structural features and global contextual dependencies enables more discriminative representation learning for large- and medium-scale vessels.

Compared with some traditional baseline CNN models such as VGG, LeNet, and ResNet50, the recognition accuracy of the proposed model (83.45%) is slightly lower for the Fishing category. However, when considering all three categories jointly, the proposed model exhibits a more balanced classification performance, with consistently high accuracies across different vessel types. In contrast, several baseline models, such as Lenet, Swin-T, and ConvNeXt-T, show relatively larger performance variations among categories, while ViT exhibits a more uniform but overall lower classification performance across all three classes.

**Figure 6:**
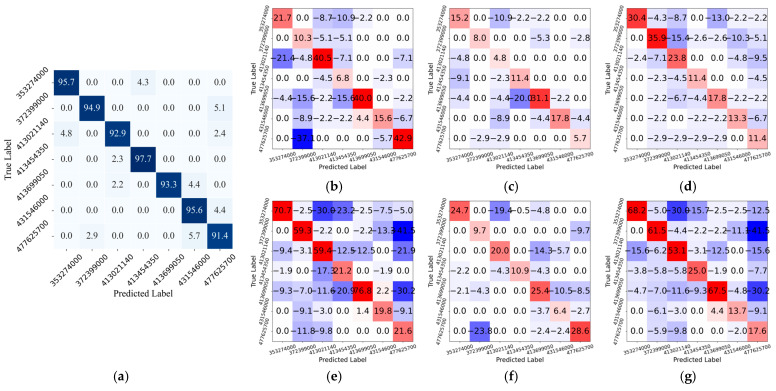
Confusion matrix of the proposed HLGF-Net on the measured dataset and the corresponding difference heatmaps compared with baseline models: (**a**) Confusion matrix of the proposed model. (**b**–**g**) Normalized confusion matrix difference heatmaps between the proposed model and VGG, ResNet50, LeNet, Swin-T, ViT, and ConvNeXt-T, respectively. Positive and negative values indicate the increase and decrease in classification probability of the proposed model relative to the compared methods.

Figure 6 further evaluates the classification behavior of the proposed HLGF-Net on the Measured Dataset, which contains richer and more complex target echo information compared with the public FUSAR dataset. As shown in Figure 6a, the proposed model maintains high recognition accuracy across all seven categories, with diagonal accuracy exceeding 90% for most groups. This observation indicates that the proposed model maintains stable classification performance across different categories in complex environments.

Analysis of the difference heatmaps in Figure 6b–g reveals that all baseline models exhibit significant performance degradation across multiple vessel categories. Compared with traditional convolutional networks such as VGG, the decline is particularly evident in several fine-grained vessel categories, indicating limited capability in distinguishing classes with highly similar feature characteristics. Despite employing more advanced feature modeling strategies, modern models like Swin-T, ViT, and ConvNeXt-T still exhibit large, scattered negative regions in their difference heatmaps, indicating insufficient prediction stability. In contrast, the proposed model demonstrates consistent positive gains across most categories, demonstrating more reliable overall classification behavior and stronger robustness.

Overall, the analyses in Figure 5 and Figure 6 confirm that the proposed HLGF-Net achieves the best balance between classification accuracy, stability, and robustness across different datasets, consistently outperforming all baseline models.

### 3.5. Ablation Analysis

To investigate the individual contributions of the key components in the proposed architecture, several ablation variants were constructed by selectively removing or modifying specific modules. When only the convolutional backbone is used, the model exhibits baseline performance (test accuracy = 84.73%, F1 = 0.8467), indicating that local convolutional features alone are insufficient for capturing global semantic dependencies in complex maritime environments.

When the Transformer module is included but the CLS token is removed (No_CLS), the performance remains almost identical to the baseline (Test Accuracy = 85.50%, F1 = 0.8561). This result suggests that, without a dedicated semantic aggregation node, the Transformer cannot effectively integrate global contextual information, and thus its modeling capacity is largely suppressed.

Interestingly, when all self-attention blocks are disabled while retaining the CLS token and tokenization structure (Trans_0blocks), the model performance increases substantially to Test Accuracy = 90.08% and F1 = 0.9001. Although this variant does not employ any self-attention operation, the token-based feature reorganization and CLS-guided global fusion already enhance the semantic relationships among local convolutional features, leading to a notable improvement over the baseline.

Finally, when multi-head self-attention blocks are incorporated (proposed model), the performance is further boosted to test accuracy = 91.35% and F1 = 0.9130. The attention mechanism captures long-range dependencies more comprehensively, enabling more effective integration of local and global information.

Overall, the ablation analysis highlights the contribution of each architectural component to the final recognition performance. The CLS token plays a central role in global semantic aggregation, the tokenization structure fundamentally strengthens the feature representation, and the self-attention mechanism provides additional performance gains by modeling long-range contextual relationships. Together, these components work synergistically to achieve the best recognition performance in challenging maritime scenarios.

## 4. Discussion

Recent research on maritime vessel recognition has primarily focused on multiscale convolutional feature modeling or global semantic modeling based on Transformers. However, few studies have successfully integrated local structural features with layer-wise deepening global dependencies within a unified framework. Compared to traditional CNNs, which heavily rely on local receptive fields, and pure Transformer networks that exhibit unstable performance under small sample conditions, the proposed HLGF-Net offers a more balanced solution. By mapping local features extracted from VGG into serialized patches and feeding them into a stacked Transformer encoder, the model progressively integrates local structural information with global semantic dependencies through multilayer self-attention interactions. This establishes a hierarchical local–global feature modeling mechanism, demonstrating enhanced robustness under conditions of maritime clutter interference, low signal-to-noise ratio, and limited samples. Comparative experiments against models such as VGG, ResNet50, ViT, Swin-T, and ConvNeXt-T demonstrate that HLGF-Net achieves optimal performance in recognition accuracy, generalization stability, and resistance to low-noise degradation. This further validates the proposed model’s significant advantages in complex maritime vessel target recognition tasks.

Despite these improvements, the model still relies on pretrained convolutional backbones and exhibits a higher parameter count than lightweight alternatives, which may limit deployment on resource-constrained maritime platforms. In addition, the current study focuses on single-frame SAR or processed echoes, and it has not yet integrated temporal information, Doppler signatures, or multimodal fusion. The measured dataset, though representative, remains limited in diversity. Future work will explore radar-specific or self-supervised pretraining to reduce domain gaps, develop lightweight variants for onboard applications, and extend the hierarchical fusion framework to temporal, multiangle, or multimodal data sources to further enhance recognition stability in complex maritime environments.

## 5. Conclusions

In conclusion, this paper proposed a hierarchical local–global feature fusion network for robust maritime target recognition in complex sea clutter environments. By progressively integrating unified local structural representations with global semantic dependencies through stacked Transformer layers, the proposed method effectively enhances feature discrimination under cluttered and low-SNR conditions. Experimental results on both a public SAR dataset and measured maritime data demonstrate that the proposed model achieves superior recognition accuracy and robustness compared with representative baseline models, while maintaining competitive inference efficiency. These findings indicate that the proposed model provides an effective and practical solution for maritime target recognition under challenging operational conditions.

## Figures and Tables

**Table 1:** Specific information on the measured dataset.

Categories	Length Overall/m	Beam/m	Draft/m	Sample Size (Statistics)
General Cargo (353274000)	153	24	9.2	200
Vehicle Carrier A (372399000)	200	32	9.0	205
Vehicle Carrier B (431546000)	180	32	7.7	200
Vehicle Carrier C (477625700)	179	32	7.6	219
Rescue Vessel (413021140)	99	15	6.0	195
Container ship (413454350)	136	23	7.8	254
Dredger (413699050)	84	13	3.9	205

**Table 2:** Parameter evaluation across different models.

Model	Final Test Accuracy	F1 Score	Parameter Sizes (M)	FLOPS	Inference Time per Sample (ms)	Training Time per Sample (ms)
Lenet	58.02%	0.4780	5.41 M	55.56 MMac	0.635	2.155
Resnet50	86.77%	0.8683	25.56 M	4.13 GMac	2.214	8.323
VGG	84.73%	0.8467	119.57 M	15.52 GMac	3.965	6.821
ViT	82.44%	0.8235	85.80 M	16.87 GMac	23.931	5.395
Proposed	91.35%	0.9130	155.21 M	31.66 GMac	3.902	12.157
Swin-T	86.77%	0.8667	27.52 M	2.19 GMac	3.368	10.525
ConvNeXt-T	86.73%	0.8681	27.82 M	4.46 GMac	2.859	18.480

## Data Availability

The datasets presented in this article are not readily available because the data are part of an ongoing study.
